# *KMT2C/D* mutations in newly diagnosed acute myeloid leukaemia: Clinical features, genetic co‐occurrences and prognostic significance

**DOI:** 10.1002/ctm2.70284

**Published:** 2025-03-26

**Authors:** Wenting Wang, Miao Yang, Xue Zhang, Jiayuan Chen, Shaowei Qiu, Bingcheng Liu, Yingchang Mi, Jianxiang Wang, Hui Wei

**Affiliations:** ^1^ State Key Laboratory of Experimental Hematology, National Clinical Research Center for Blood Diseases, Haihe Laboratory of Cell Ecosystem Institute of Hematology & Blood Diseases Hospital, Chinese Academy of Medical Sciences & Peking Union Medical College Tianjin China; ^2^ Tianjin Institutes of Health Science Tianjin China

1

To the Editor:

Acute myeloid leukaemia (AML) is a diverse and complex category of malignant disease with poor outcomes. Despite advancements in prognostication and treatment strategies, the molecular landscape of AML remains complex and is not fully understood. Epigenetic factors are acknowledged as crucial in tumour development and progression.^1^ The *KMT2* (histone‐lysine N‐methyltransferase 2) family encompasses six key proteins: *KMT2A/B*, *KMT2C/D*, *KMT2F* and *KMT2G*, which is most notably associated with AML.^2^ *KMT2A* is associated with *KMT2A*‐rearranged leukaemia.^3^ *KMT2B* is identified as a hotspot for rearrangements.^4^ *KMT2C/D* mutations occurred frequently in various malignancies.^5^ However, the clinical characteristics of *KMT2C/D* mutations in AML remain poorly defined. We found that *KMT2C* and *KMT2D* mutations are relatively rare and mutually exclusive in newly diagnosed AML, with *KMT2C* mutations enriched in *CEBPA*‐mutated and *KMT2D* in *NPM1*‐mutated AML subtypes, respectively. In general, *KMT2C^MUT^* and *KMT2C^WT^*, as well as *KMT2D^MUT^* and *KMT2D^WT^* AML, exhibit distinct mutational spectrums, similar clinical characteristics and survival outcomes.

We reviewed 1935 AML patients who underwent next‐generation sequencing (NGS) analyses between 2015 and 2024. Of these, 1050 were eligible for *KMT2C* analysis and 1777 for *KMT2D* analysis. The *KMT2C* mutation rate was 1.90% (20/1050), consistent with previous studies.^6^, ^7^ The *KMT2D* mutation rate was 1.41% (25/1777), lower than that reported in a small‐sample study.^8^ No patient had concurrent *KMT2C* and *KMT2D* mutations. Characteristics of AML patients with and without *KMT2C/D* mutation are presented in Table 1. Clinical characteristics did not differ significantly between wild‐type and *KMT2C/D*‐mutated AML patients, except that *KMT2D^WT^* patients had higher haemoglobin levels than *KMT2D^MUT^* patients (*p *< .001). Among *KMT2C* mutated patients, nine (45%) were male and their median age was 43.9 years. For AML classification, 10 of 20 patients were classified as AML with *CEBPA* mutation. Patients with *KMT2D* mutations included 11 (44%) males, with a median age of 42.0, and 11 (44%) were recognised as AML with *NPM1* mutation. Patients with *KMT2C* and *KMT2D* mutations were enriched by AML with *CEBPA* and *NPM1* mutations, respectively. Additionally, most patients had normal karyotypes, including 50% of *KMT2C^MUT^* patients and 72% of *KMT2D^MUT^* patients.

**TABLE 1:** Characteristics of newly diagnosed AML patients with and without *KMT2C* /*D* mutation in our cohort

	*KMT2C^MUT^* (*n* = 20)	*KMT2C^WT^* (*n* = 1030)		*KMT2D^MUT^* (*n* = 25)	*KMT2D^WT^*	
Characteristic			*p1*		(*n* = 1752)	*p2*
Age (years)	43.9 (16–63)	41.9 (14–79)	.598	42.0 (15–71)	41.9 (13–82)	.976
Gender			.518			.428
Male (*N*)	9 (45.0%)	551 (53.5%)		11 (44.0%)	946 (54.0%)	
Female (*N*)	11 (55.0%)	479 (46.5%)		14 (56.0%)	806 (46.0%)	
Peripheral blood						
WBC (10^9^/L)	23.4 (1.0–106.2)	30.8 (.6–317.1)	.312	21.4 (1.1–132.9)	33.0 (.5–404.1)	.093
Haemoglobin (g/L)	87.8 (57.0–135.0)	84.4 (40.0–144.0)	.552	70.0 (47.0–97.0)	86.1 (34.0–158.0)	<.001
Platelets (10^9^/L)	58.0 (8.0–244.0)	54.8 (2.0–376.0)	.811	60.0 (16.0–176.0)	61.8 (2.0–1852.0)	.817
AML classification			.127			.022
AML with *RUNX1::RUNX1T1* fusion	1	154		4	284	
AML with *CBFβ::MYH11* fusion	0	93		2	132	
AML with *DEK::NUP214* fusion	0	2		0	8	
AML with *BCR::ABL1* fusion	0	3		0	8	
AML with *KMT2A* rearrangement	1	63		0	104	
AML with *NPM1* mutation	2	192		11	317	
AML with *CEBPA* mutation	10	211		0	340	
AML with myelodysplasia related	4	148		5	248	
AML defined by differentiation	2	164		3	311	
Chromosome karyotype			.011			.410
Normal karyotype	10 (50.0%)	460 (44.7%)		18 (72.0%)	795 (45.4%)	
T(8;21)	1 (5.0%)	154 (15.0%)		4 (16.0%)	279 (15.9%)	
Inv(16)	0 (0%)	84 (8.2%)		1 (4.0%)	122 (7.0%)	
T(v;11q23.3)	1 (5.0%)	36 (3.5%)		0 (0%)	56 (3.1%)	
Del(7)/‐7/abn(17p)	2 (10.0%)	14 (1.4%)		0 (0%)	30 (1.7%)	
Del(5q)	1 (5.0%)	1 (.1%)		0 (0%)	5 (.3%)	
Del(9q)	1 (5.0%)	19 (1.8%)		0 (0%)	30 (1.7%)	
Trisomy	1 (5.0%)	57 (5.5%)		1 (4.0%)	112 (6.4%)	
Other abnormal karyotype	5 (25.0%)	141 (13.7%)		0 (0%)	242 (13.8%)	
ND	0 (0%)	64 (6.2%)		1 (4.0%)	81 (4.6%)	

*Note*: p1: *KMT2C^MUT^* vs. *KMT2C^WT^*; *p2*: *KMT2D^MUT^* vs. *KMT2D^WT^*.

Abbreviations: ND, not detected; WBC, white blood cell count.

We identified 22 mutation sites in the *KMT2C* gene (Figure 1A) and 30 in the *KMT2D* gene (Figure 1B). In all 95.5% of *KMT2C* mutations were nonsense and frameshift, consistent with the COSMIC database analysis by Rao et al.^5^ The PHD domain was the most frequently mutated region in *KMT2C*, with 27.3% (6/22) of mutations located there, of which 83.3% (5/6) were nonsense. In contrast, *KMT2D* mutations were dispersed without clustering in specific exons or domains. We also analysed co‐mutations in *KMT2C/D* mutant patients. A total of 110 (median five mutations per patient) and 128 (median five mutations per patient) mutations had been found in *KMT2C* and *KMT2D* mutated AML, respectively. All *KMT2C/D* mutations were accompanied by additional gene mutations. *CEBPA* (*n* = 10, 50.0%) was the most frequent co‐mutation with *KMT2C*, followed by *NRAS* (*n* = 7, 35.0%), *GATA2* (*n* = 5, 25.0%), *FLT3* (*n* = 5, 25.0%). Garg et al. reported that *KMT2C* co‐mutates with *FLT3*.^9^ In addition, compared with *KMT2C^WT^* AML, *KMT2C^MUT^* AML had significantly higher frequencies of *CEBPA* (50.0% vs. 20.6%, *p* = .004), *GATA2* (25.0% vs. 6.1%, *p *= .007) and *BCOR* (20.0% vs. 4.9%, *p* = .016) mutations (Figure 1C). Besides, all *CEBPA* mutations were *CEBPA^bZIP^* (Figure 1D). Correspondingly, *FLT3* (*n* = 13, 52.0%) was the most frequent co‐occurred mutation in *KMT2D^MUT^* AML, followed by *NPM1* (*n* = 11, 44.0%) and *IDH2* (*n* = 7, 28.0%). Compared with *KMT2D^WT^* AML, *KMT2D^MUT^* patients were more likely to co‐occur with *FLT3* (52.0% vs. 28.1%, *p* = .016) and *NPM1* (44.0% vs. 19.2%, *p* = .004) mutations (Figure 1E,F).

**FIGURE 1:**
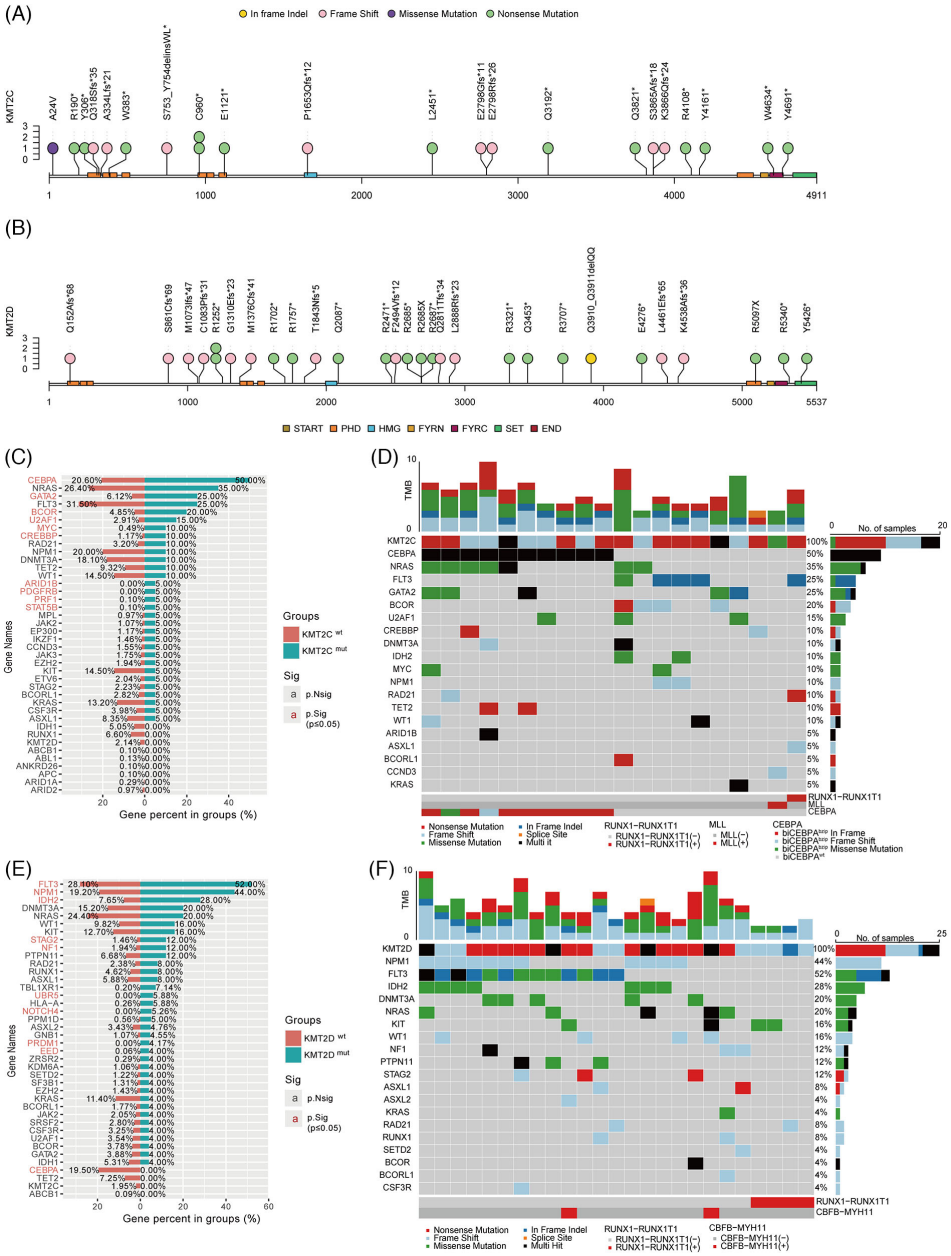
*KMT2C/D* mutations in AML. The distribution of *KMT2C* (A) and *KMT2D* (B) mutations, which were identified in our cohort, on the protein. Bar chart of additional gene mutation distribution in *KMT2C^WT^* and *KMT2C^MUT^* groups (C), and additional gene mutation distribution in *KMT2D^WT^* and *KMT2D^MUT^* groups (E), sorted according to the mutation rate in a mutated group, and the percentage of each gene mutation is shown. (D) Mutation oncoplot of the 20 AML patients with *KMT2C* mutation. (F) Mutation oncoplot of the 25 AML patients with *KMT2D* mutation.

Further, we compared the characteristics of different mutation statuses of *KMT2C/D* in the *CEBPA^bZIP^* (Table S1) and *NPM1* (Table S2) subgroups, respectively. In both subgroups, clinical characteristics were similar between wild‐type and *KMT2C/D*‐mutated AML patients, except for the higher haemoglobin in *KMT2D^WT^* patients (*p* = .035). In the *CEBPA^bZIP^* AML cohort, *KMT2C^MUT^* AML were more likely to have co‐mutations of *NRAS* (50.0% vs. 21.1%, *p* = .05) than *KMT2C^WT^*. In the *NPM1* ^MUT^ subgroup, the prevalence of *IDH2* co‐mutations was significantly higher in *KMT2D^MUT^* AML compared with *KMT2D^WT^* AML (63.6% vs. 18.7%, *p* = .002).

Finally, we examined whether *KMT2C/D* mutations influence the prognosis of AML. There was no difference in overall survival (OS) or event‐free survival (EFS) between *KMT2C^WT^* and *KMT2C^MUT^* AML patients. The 1‐year OS rates were 83.8% and 94.7% for *KMT2C^WT^* and *KMT2C^MUT^* groups (HR:. 69, 95% confidence interval [CI]:. 22–2.15, *p* = .52; Figure 2A), respectively. The 1‐year EFS rate in *KMT2C^WT^* and *KMT2C^MUT^* groups were 50.5% and 63.8% (HR:. 64, 95% CI:. 32–1.28, *p* = .2; Figure 2B), respectively. Although no difference in OS was observed between *CEBPA^bZIP^*/*KMT2C^WT^* and *CEBPA^bZIP^*/*KMT2C^MUT^* patients (Figure 2C), the *CEBPA^bZIP^*/*KMT2C^MUT^* patients exhibited superior EFS, achieving 1‐year EFS rate of 90.0% in contrast to 55.4% in the *CEBPA^bZIP^*/*KMT2C^WT^* patients (HR:. 15; 95% CI:. 02–1.06, *p* = .028; Figure 2D). Multivariate analysis also demonstrated that *KMT2C* mutation in *CEBPA^bZIP^* patients was associated with better EFS (HR:. 102, 95% CI:. 013–.766, *p *= .026) but not OS (Table S3). *KMT2D^MUT^* and *KMT2D^WT^* groups exhibited similar outcomes with 1‐year OS rates at 81.5% and 88.4% (HR:. 78, 95% CI:. 29–2.09, *p* = .62; Figure 2E), 1‐year EFS rate at 49.5% and 49.0% (HR: 1.09, 95% CI:. 62–1.93, *p* = .76; Figure 2F), respectively. Moreover, for *NPM1^MUT^* patients, *KMT2D* mutation did not affect EFS or OS, as shown by Kaplan–Meier survival analysis (Figure 2G,H) and multivariate analysis (Table S4). Additionally, we investigated the impact of *KMT2C/D* mutations on survival outcomes across different NCCN risk groups, yet found no significant effects in the favourable, intermediate or adverse risk groups (Figures S1 and S2). However, the effect of *KMT2C/D* on the prognosis of AML needs to be further explored and verified by more studies with larger samples.

**FIGURE 2:**
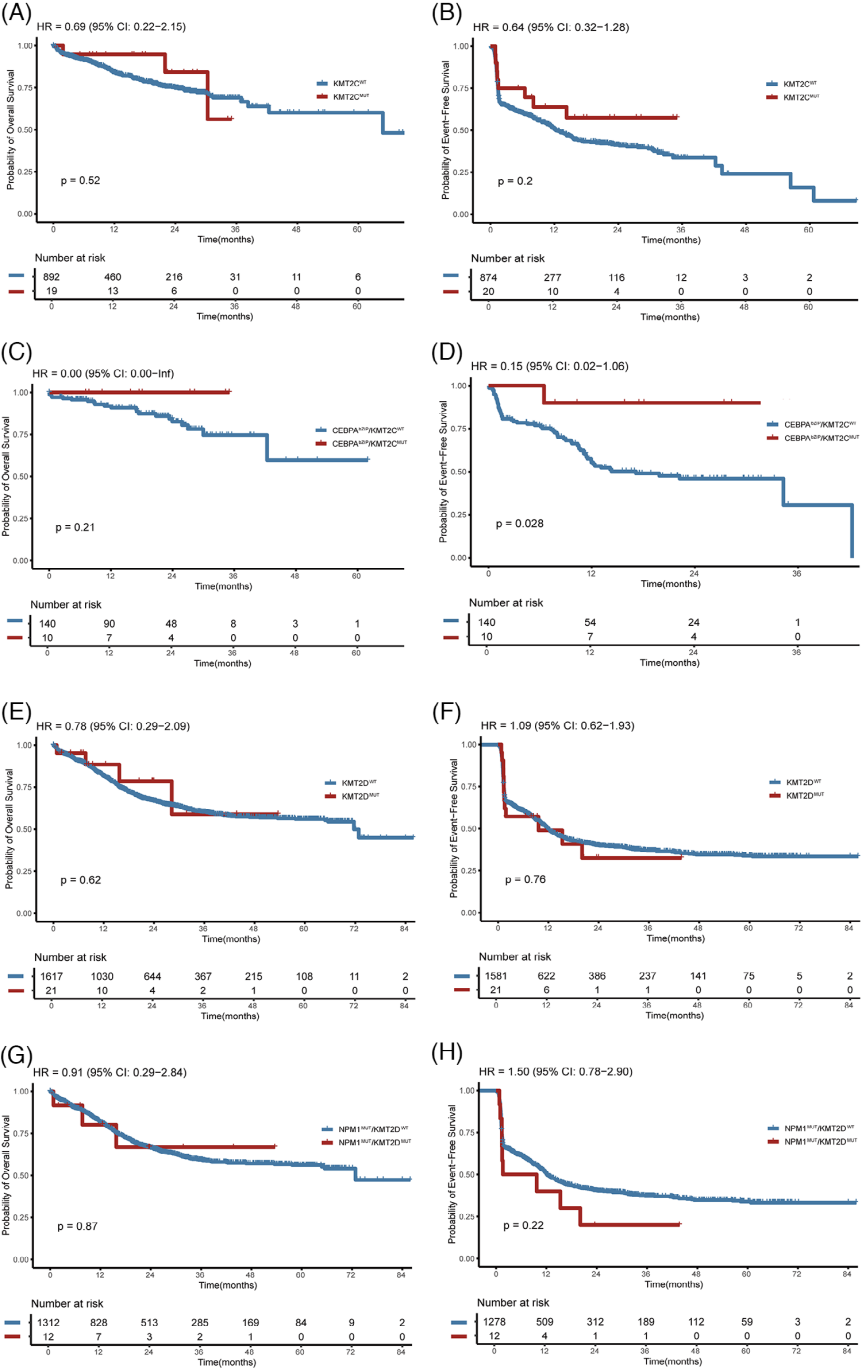
The overall survival (OS) (A) and event‐free survival (EFS) (B) of *KMT2C^WT^* and *KMT2C^MUT^* groups in our cohort, and OS (C) and EFS (D) of *KMT2C^WT^* and *KMT2C^MUT^* groups in the *CEBPA^bZIP^* subgroup. The OS (E) and EFS (F) of *KMT2D^WT^* and *KMT2D^MUT^* groups in our cohort, and OS (G) and EFS (H) of *KMT2D^WT^* and *KMT2D^MUT^* groups in the *NPM1^MUT^* subgroup.

## AUTHOR CONTRIBUTIONS

HW and JW participated in concept design. MY, XZ, and WW were involved in data collection and analysis, drafting and revising the manuscript. JC, SQ, BL, and YM were responsible for interpreting the results. All authors have read and approved the final manuscript. 

## FUNDING INFORMATION

National Key Research and Development Program of China, Grant Number: 2023YFC2508900; National Natural Science Foundation of China, Grant Number: 82370183; CAMS Innovation Fund for Medical Sciences, Grant Number: 2023‐I2M‐2‐007; Tian Jin Natural Science Foundation, Grant Number: 23JCZXJC00310; Haihe Laboratory of Cell Ecosystem Innovation Fund, Grant Number: 22HHXBSS00040; Beijing Xisike Clinical Oncology Research Foundation, Grant Number: Y‐SYBLD2022ZD‐0031

## ETHICS STATEMENT

This research was approved by the ethical committee in the Institute of Hematology and Blood Diseases Hospital, and all procedures were in accordance with the Helsinki Declaration. Written informed consent was obtained from each participant.

## Supporting information



Supporting Information

Supporting Information

Supporting Information

Supporting Information

Supporting Information

Supporting Information

Supporting Information

Supporting Information

Supporting Information
