# Supplementary material for: KMT2C/D mutations in newly diagnosed acute myeloid leukaemia: Clinical features, genetic co‐occurrences and prognostic significance
Source: Clin Transl Med. 2025 Mar 26;15(4):e70284. doi: 10.1002/ctm2.70284 (PMC11946544; doi:10.1002/ctm2.70284)
Supplement: Supplementary file 1 — Supporting Information [file CTM2-15-e70284-s002.docx]

**Disscussion**

As we know, it was the first time that we conducted an in-depth analysis of *KMT2C* and *KMT2D* mutations in AML. We found that *KMT2C* and *KMT2D* mutations are relatively rare and mutually exclusive in AML. Our study demonstrated that *KMT2C^MUT^* and *KMT2D^MUT^* AML patients were enriched by AML with *CEBPA* and *NPM1* mutation, respectively. In additions, we found that the *CEBPA^bZIP^*/*KMT2C^MUT^* patients exhibited superior EFS compared with *CEBPA^bZIP^/KMT2C^WT^* AML patients.

We found that *KMT2C* and *KMT2D* mutations are relatively rare in AML. The mutation rate of *KMT2C* was 1.90%, similar to the mutation rate found in previous studies [1, 2]. However, few studies have systematically investigated the mutation rate of *KMT2D* in AML. In the study by Meena et al. [3], an 8% *KMT2D* mutation rate was reported in a small cohort of 25 AML patients. In contrast, our study, which includes a larger patient population, we observed a lower *KMT2D* mutation rate of 1.41%. The discrepancy may due to sample size and demographics. In addition, we found that *KMT2C* and *KMT2D* mutations appear to cluster in specific AML subtypes. *KMT2C* mutated patients were enriched by AML with *CEBPA* mutation, and more precisely, it co-occurs with the *CEBPA^bZIP^* mutation, a known favorable prognostic factor. A study by Garg et al. has reported that *KMT2C* co-mutates with *FLT3* [4]. In our study, there were 5 patients (25%) in *KMT2C^MUT^* AML harbored the *FLT3* mutation, one of the common mutations in *KMT2C^MUT^* AML in our cohort. Besides, in study by Halik et al., The *KMT2C* mutation rate is 10% in AML with abnormal chromosome 7. However, we observed that only 2 of the 40 patients with abnormal chromosome 7 in our cohort had *KMT2C* mutations. Our study revealed *KMT2D* mutation was clustered in AML with *NPM1* mutation. This finding is consistent with the observations of Meena et al. who found that 2 patients had *KMT2D* mutations, and that both occurred in AML with *NPM1* mutation [3].

Our study also provided a detailed analysis of the mutational spectrum of *KMT2C* and *KMT2D*. We found most *KMT2C/D* mutations were nonsense and frameshift, suggesting a potential loss of function for the affected proteins. This finding is in agreement with analysis of the COSMIC database by Rao et al., which also reported a high prevalence of nonsense and frameshift mutations in *KMT2C/D* across multiple cancer types [5]. However, a study by Halik et al. found that in AML with chromosome 7 abnormalities, 3/4 of the *KMT2C* mutations are missense. Our study did not show the clustering of mutations in the SET domain reported by Rao et al. [6]. We found *KMT2C* mutations mainly located in PHD domains and *KMT2D* mutations were scattered across the gene. This could be related to the fact that we focused on AML rather than pan-cancer. These discrepancies may be attributed to the distinct characteristics of each study's cohort, our study was specifically focused on the broader spectrum of AML, in contrast to the other studies that either adopted a pan-cancer perspective or concentrated on AML with chromosome 7 abnormalities.

Our study still has some limitations. Firstly, our study was a retrospective study. Secondly, the sample size of our study was not large enough, coupled with the low frequency of *KMT2C*/*D* mutations, resulting in a small number of patients with *KMT2C/D* mutations that were finally available for analysis. Consequently, future large-scale, prospective clinical studies are warranted to reveal and confirm the impact of *KMT2C/D* mutations on AML.

**Reference**

1. Dolnik A, Engelmann JC, Scharfenberger-Schmeer M, Mauch J, Kelkenberg-Schade S, Haldemann B, et al. Commonly altered genomic regions in acute myeloid leukemia are enriched for somatic mutations involved in chromatin remodeling and splicing. Blood. 2012;120(18):E83-E92.

2. Ley TJ, Miller C, Ding L, Raphael BJ, Mungall AJ, Robertson A, et al. Genomic and epigenomic landscapes of adult de novo acute myeloid leukemia. The New England journal of medicine. 2013;368(22):2059-74.

3. Meena JP, Pathak N, Gupta AK, Bakhshi S, Gupta R, Makkar H, et al. Molecular evaluation of gene mutation profiles and copy number variations in pediatric acute myeloid leukemia. Leukemia research. 2022;122:106954.

4. Garg M, Nagata Y, Kanojia D, Mayakonda A, Yoshida K, Haridas Keloth S, et al. Profiling of somatic mutations in acute myeloid leukemia with FLT3-ITD at diagnosis and relapse. Blood. 2015;126(22):2491-501.

5. Rao RC, Dou Y. Hijacked in cancer: the KMT2 (MLL) family of methyltransferases. Nature reviews Cancer. 2015;15(6):334-46.

6. Halik A, Tilgner M, Silva P, Estrada N, Altwasser R, Jahn E, et al. Genomic characterization of AML with aberrations of chromosome 7: a multinational cohort of 519 patients. Journal of hematology & oncology. 2024;17(1):70.
