# Supplementary figures and images for: KMT2C/D mutations in newly diagnosed acute myeloid leukaemia: Clinical features, genetic co‐occurrences and prognostic significance
Source: Clin Transl Med. 2025 Mar 26;15(4):e70284. doi: 10.1002/ctm2.70284 (PMC11946544; doi:10.1002/ctm2.70284)

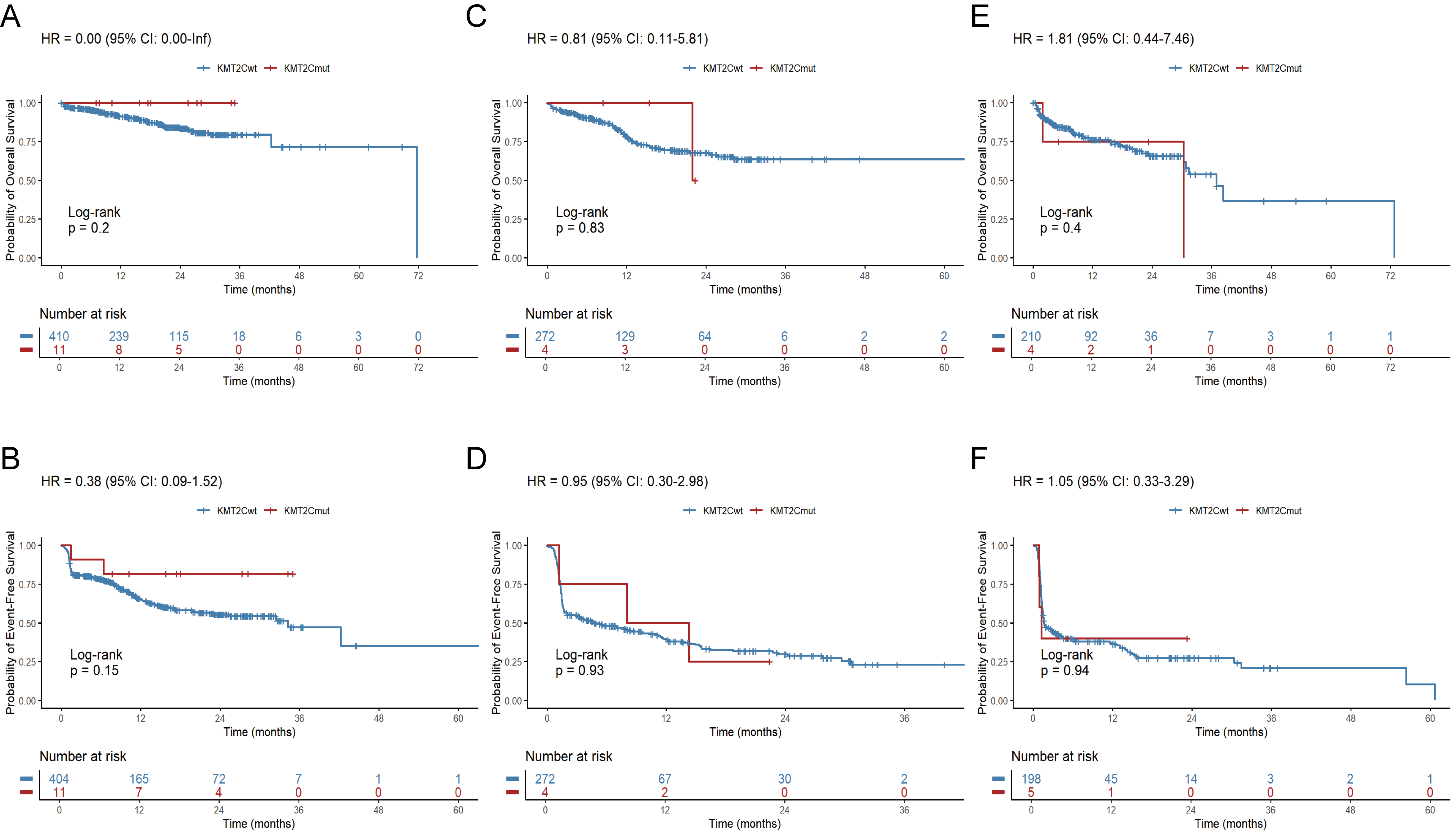

Supplement: Supplementary file 2 — Supporting Information [file CTM2-15-e70284-s001.tif]

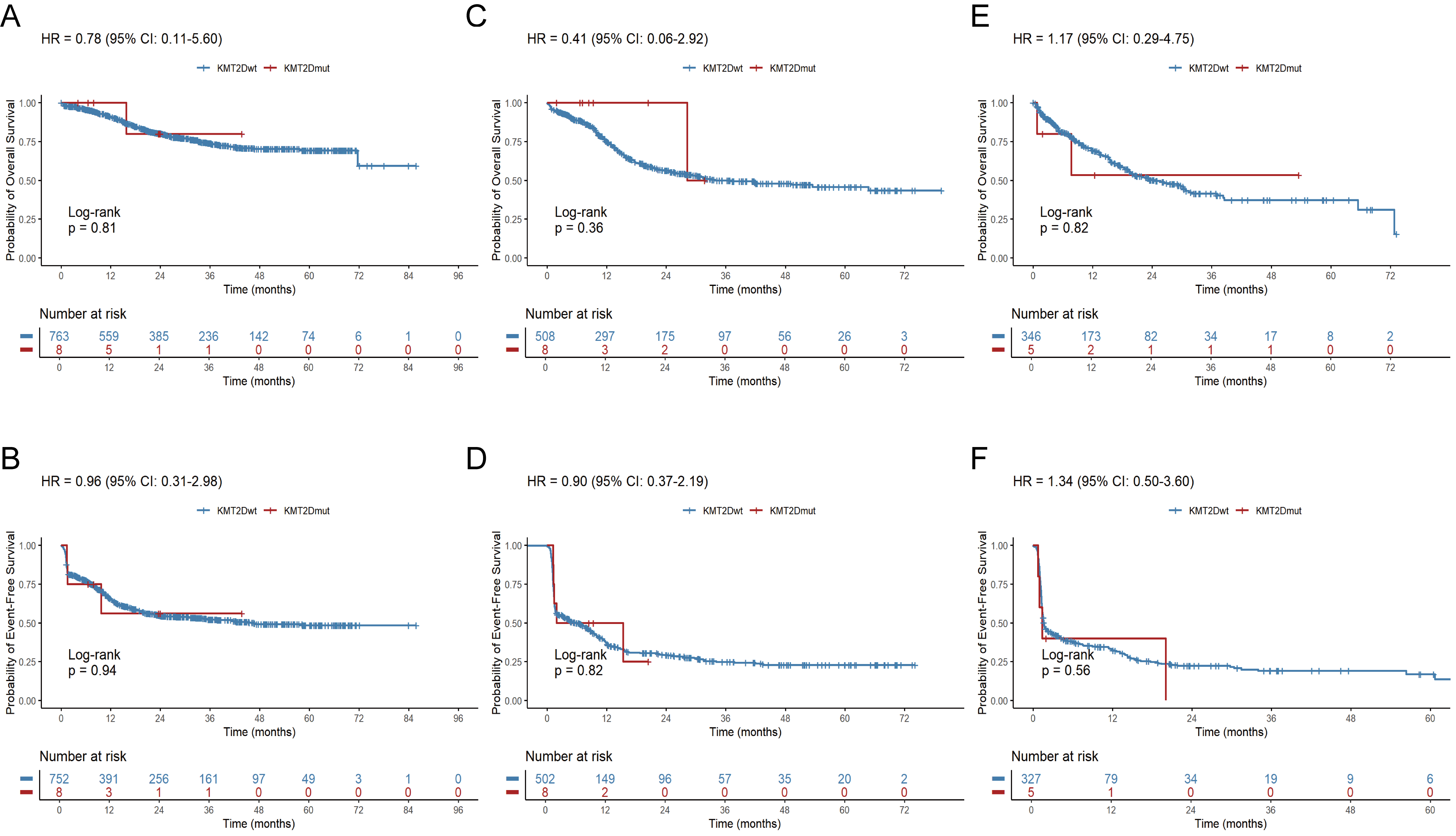

Supplement: Supplementary file 3 — Supporting Information [file CTM2-15-e70284-s007.tif]
