# Supplementary material for: KMT2C/D mutations in newly diagnosed acute myeloid leukaemia: Clinical features, genetic co‐occurrences and prognostic significance
Source: Clin Transl Med. 2025 Mar 26;15(4):e70284. doi: 10.1002/ctm2.70284 (PMC11946544; doi:10.1002/ctm2.70284)
Supplement: Supplementary file 4 — Supporting Information [file CTM2-15-e70284-s004.docx]

**Methods**

**Patients’ selection**

We retrospectively collected data from 1935 AML patients at our center between January 2015 and February 2024. The inclusion and exclusion criteria include: 1) newly-diagnosed AML. 2) exclusion of acute promyelocytic leukemia (APL). 3) NGS was performed. Patients without mutation testing for *KMT2C* or *KMT2D* were excluded from the respective analyses. This research received approval from the hospital's ethical review board and was carried out in compliance with the principles of the Declaration of Helsinki. All patients received written informed consent for treatment and genetic testing.

**Treatment**

Patients who met the criteria for intensive therapy were treated with cytarabine and daunorubicin (DA), or in combination with homoharringtonine (HAD). Upon achieving complete remission, they continued with 3 to 4 cycles of high or intermediate dose cytarabine for consolidation therapy. For those not suitable for such intensive treatment, a low-intensity treatment plan involving hypomethylating agents (HMA) was provided. All individuals in this study were advised to consider allogeneic hematopoietic stem cell transplantation as part of their treatment journey.

**Gene mutations**

All detected genes were strongly associated with hematological malignancies. Bone marrow mononuclear cell-derived DNA was used for targeted sequencing analysis. The NGS panel used in this study varied over time, reflecting advancements in genetic testing and the evolving understanding of the genetic landscape of AML. The specific genes included in each panel are detailed in Supplementary Table 5. In summary, the panels included key genes such as *KMT2C* and *KMT2D*, as well as other genes relevant to AML pathogenesis and prognosis.

Following enzymatic fragmentation, adapters containing molecular barcodes (for sample identification), universal PCR primers, flow cell hybridization sequences, and sequencing initiation sites were ligated to DNA fragments. Biotinylated probes were hybridized to capture target regions, and streptavidin magnetic beads were employed for library enrichment. After amplification and pooling, paired-end sequencing (PE150) was performed on the Illumina NovaSeq 6000 platform. The sequencing achieved an average coverage of 98% with a mean depth of 2,000×. Strict quality control metrics were applied, with Q30 scores exceeding 85% (over 90% of data surpassing 90% Q30). All sequencing data were aligned to the GRCh37/hg19 reference genome for identification of single nucleotide variants (SNVs) and insertions/deletions (Indels).

Validation using qPCR and other orthogonal methods confirmed a limit of detection (LOD) of 2% variant allele frequency (VAF). In clinical reporting, results were interpreted comprehensively by integrating patients' medical history and disease progression status. Variant filtering criteria included: 1) Sequencing depth ≥1,000× (adjusted for regions with copy number variations [CNVs] after IGV visualization); 2) VAF ≥1%; 3) Exclusion of non-hotspot variants (those not specified in WHO, NCCN, ELN, or other authoritative guidelines) with minor allele frequency (MAF) ≥1% in population databases (ExAC, ESP6500, GnomAD). Clinical significance of variants was annotated using disease-related databases including COSMIC, ClinVar, HGMD, and dbSNP.

To ensure variant authenticity, the following additional measures were implemented: 1) Intra-batch reproducibility analysis; 2) Combined evaluation of low-frequency variants (VAF approaching 1%) considering technical reproducibility and clinical relevance; 3) IGV-based visualization to inspect read distribution patterns and regional coverage depth; 4) Manual verification of read counts and mutation localization to eliminate pseudogene-derived artifacts in homologous regions.

**Statistical analysis**

The overall survival (OS) and event-free survival (EFS) were estimated using the Kaplan-Meier method and their differences were statistically assessed by the Log-rank test. The OS period was defined as the time interval from the date of diagnosis until the date of death or last follow-up. EFS period was calculated as the time between the date of diagnosis and the time of induction failure after 1 course induction, relapse, death or last follow-up. A complete remission (CR) was determined when the bone marrow aspirate showed less than 5% blasts upon morphological examination and there was an absence of detectable extramedullary disease. The chi-square was utilized for comparing continuous variables, while the t-test was employed to assess the significance of categorical variable differences. For small sample sizes, the Fisher's exact test was applied. The statistical tests were conducted with a two-tailed significance level of 0.05. Analyses were performed on the R software platform (R version 4.4.1; R Foundation for Statistical Computing, Vienna, Austria).
