# Supplementary material for: KMT2C/D mutations in newly diagnosed acute myeloid leukaemia: Clinical features, genetic co‐occurrences and prognostic significance
Source: Clin Transl Med. 2025 Mar 26;15(4):e70284. doi: 10.1002/ctm2.70284 (PMC11946544; doi:10.1002/ctm2.70284)
Supplement: Supplementary file 5 — Supporting Information [file CTM2-15-e70284-s005.docx]

**Table S1.** Characteristics of AML patients with *CEBPA^bZIP^* mutation according to *KMT2C* mutational status.

|  | *CEBPA^bZIP^* /*KMT2C^WT^* | *CEBPA^bZIP^* /*KMT2C^MUT^* | P | N |
| --- | --- | --- | --- | --- |
|  | N=152 | N=10 |  |  |
| Age (years) | 40.7 (14-73) | 43.6 (16-58) | 0.486 | 162 |
| Gender |  |  | 0.342 | 162 |
| Male | 86 (56.6%) | 4 (40.0%) |  |  |
| Female | 66 (43.4%) | 6 (60.0%) |  |  |
| Peripheral blood |  |  |  |  |
| WBC (10^9^/L) | 43.4 (2.0-424.0) | 31.6 (2.4-106.2) | 0.398 | 162 |
| Hemoglobin (g/L) | 93.4 (45.0-153.0) | 84.9 (57.0-134.0) | 0.313 | 162 |
| Platelets (10^9^/L) | 36.4 (3.0-160.0) | 37.5 (19.0-91.0) | 0.886 | 162 |
| Gene mutations(N) |  |  |  |  |
| *NRAS* | 32 (21.1%) | 5 (50.0%) | 0.050 | 162 |
| *CSF3R* | 18 (11.8%) | 1 (10.0%) | 1.000 | 162 |
| *GATA2* | 36 (23.7%) | 3 (30.0%) | 0.705 | 162 |
| *TET2* | 22 (14.5%) | 2 (20.0%) | 0.644 | 162 |
| *WT1* | 43 (28.3%) | 1 (10.0%) | 0.289 | 162 |

WBC, white blood cell count.
