# Supplementary material for: KMT2C/D mutations in newly diagnosed acute myeloid leukaemia: Clinical features, genetic co‐occurrences and prognostic significance
Source: Clin Transl Med. 2025 Mar 26;15(4):e70284. doi: 10.1002/ctm2.70284 (PMC11946544; doi:10.1002/ctm2.70284)
Supplement: Supplementary file 6 — Supporting Information [file CTM2-15-e70284-s008.docx]

**Table S2.** Characteristics of AML patients with *NPM1* mutation according to *KMT2D* mutational status.

|  | *NPM1^MUT^/KMT2D^WT^*  N=337 | *NPM1^MUT^/KMT2D^MUT^*  N=11 | P | N |
| --- | --- | --- | --- | --- |
| Age (years) | 46.7(14-75) | 44.5 (15-71) | 0.657 | 348 |
| Gender |  |  | 0.357 | 348 |
| Male | 155 (46.0%) | 3 (25.0%) |  |  |
| Female | 182 (54.0%) | 8(75.0%) |  |  |
| Peripheral blood |  |  |  |  |
| WBC (10^9^/L) | 43.9 (0.6-301.0) | 29.3 (1.5-75.8) | 0.145 | 348 |
| Hemoglobin (g/L) | 84.7 (26.0-164.0) | 73.5 (55.0-97.0) | 0.035 | 348 |
| Platelets (10^9^/L) | 78.7 (7.0-486.0) | 65.2 (22.0-158.0) | 0.267 | 348 |
| Gene mutations(N) |  |  |  |  |
| *DNMT3A* | 153 (45.4%) | 3 (27.3%) | 0.357 | 348 |
| *FLT3* | 189 (56.1%) | 7 (63.6%) | 0.762 | 348 |
| *IDH2* | 63 (18.7%) | 7 (63.6%) | 0.002 | 348 |
| *PRDM1* | 0 (0.00%) | 1 (9.1%) | 0.039 | 279 |
| *STAG2* | 0 (0.00%) | 1 (9.1%) | 0.034 | 328 |
| *ZRSR2* | 0 (0.00%) | 1 (9.1%) | 0.032 | 341 |

WBC, white blood cell count.
