# Supplementary material for: KMT2C/D mutations in newly diagnosed acute myeloid leukaemia: Clinical features, genetic co‐occurrences and prognostic significance
Source: Clin Transl Med. 2025 Mar 26;15(4):e70284. doi: 10.1002/ctm2.70284 (PMC11946544; doi:10.1002/ctm2.70284)
Supplement: Supplementary file 7 — Supporting Information [file CTM2-15-e70284-s003.docx]

Supplementary Table S3. Multivariate cox analysis of prognostic factors in OS and EFS in *CEBPA^bZIP^* AML patients.

|  | Overall survival | | Event-free survival | |
| --- | --- | --- | --- | --- |
|  | HR (95%CI) | *p* | HR (95%CI) | *p* |
| Age | 1.125(1.042 - 1.215) | 0.003 | 1.021(0.989 - 1.054) | 0.199 |
| Gender | 0.305(0.046 - 2.006) | 0.217 | 0.95(0.454 - 1.986) | 0.892 |
| WBC | 1.006(0.995 - 1.018) | 0.259 | 1.003(0.998 - 1.009) | 0.24 |
| *CSF3R* mutation | 0.275(0.017 - 4.493) | 0.365 | 0.438(0.093 - 2.051) | 0.295 |
| *FLT3* mutation | 15.441(2.052 - 116.202) | 0.008 | 0.677(0.196 - 2.336) | 0.537 |
| *GATA2* mutation | 0.386(0.046 - 3.235) | 0.38 | 0.588(0.227 - 1.524) | 0.274 |
| *TET2* mutation | 1.748(0.217 - 14.054) | 0.599 | 4.329(1.47 - 12.75) | 0.008 |
| *WT1* mutation | 1.693(0.434 - 6.605) | 0.449 | 0.851(0.394 - 1.838) | 0.681 |
| *KMT2C* mutation | 0(0 - Inf) | 0.999 | 0.102(0.013 - 0.766) | 0.026 |
| Transplantation in CR1 (time- dependent) | 1.764(0.317 - 9.803) | 0.517 | 0.451(0.057 - 3.59) | 0.452 |
