# Supplementary material for: KMT2C/D mutations in newly diagnosed acute myeloid leukaemia: Clinical features, genetic co‐occurrences and prognostic significance
Source: Clin Transl Med. 2025 Mar 26;15(4):e70284. doi: 10.1002/ctm2.70284 (PMC11946544; doi:10.1002/ctm2.70284)
Supplement: Supplementary file 8 — Supporting Information [file CTM2-15-e70284-s009.docx]

Supplementary Table S4. Multivariate cox analysis of prognostic factors in OS and EFS in *NPM1^MUT^* AML patients.

|  | Overall survival | | Event-free survival | |
| --- | --- | --- | --- | --- |
|  | HR(95%CI) | *p* | HR(95%CI) | *p* |
| Age | 1.01(0.989 - 1.031) | 0.353 | 1.012(0.995 - 1.029) | 0.156 |
| Gender | 1.255(0.771 - 2.04) | 0.361 | 1.241(0.856 - 1.8) | 0.254 |
| WBC | 1.011(1.006 - 1.016) | <0.001 | 1.009(1.006 - 1.013) | <0.001 |
| *FLT3* mutation | 2.218(1.333 - 3.693) | 0.002 | 1.27(0.864 - 1.866) | 0.224 |
| *DNMT3A* mutation | 1.309(0.802 - 2.138) | 0.282 | 0.926(0.628 - 1.366) | 0.698 |
| *TET2* mutation | 0.437(0.176 - 1.084) | 0.074 | 0.787(0.407 - 1.521) | 0.476 |
| *IDH1* mutation | 1.267(0.634 - 2.532) | 0.503 | 1.393(0.771 - 2.514) | 0.272 |
| *IDH2* mutation | 0.94(0.462 - 1.911) | 0.863 | 1.196(0.739 - 1.936) | 0.466 |
| *KMT2D* mutation | 0.624(0.082 - 4.726) | 0.648 | 0.752(0.232 - 2.441) | 0.635 |
| Transplantation in CR1 (time- dependent) | 0.448(0.235 - 0.854) | 0.015 | 0.453(0.202 - 1.014) | 0.054 |
